# Technical Note: mzML and imzML Libraries for Processing Mass Spectrometry Data with the High-Performance Programming Language Julia

**DOI:** 10.1021/acs.analchem.3c05853

**Published:** 2024-03-01

**Authors:** Ignacio Rosas-Román, Héctor Guillén-Alonso, Abigail Moreno-Pedraza, Robert Winkler

**Affiliations:** † Universidad de Guanajuato, División de Ciencias e Ingenierías, Loma del Bosque 103, Lomas del Campestre, 37150 León, Guanajuato, Mexico; ‡ Center for Research and Advanced Studies (CINVESTAV) Irapuato, UGA-Langebio, Km. 9.6 Libramiento Norte Carr. Irapuato-León, 36824 Irapuato, Guanajuato, Mexico; ¶ Department of Biochemical Engineering, National Technological Institute, 38010 Celaya, Guanajuato, Mexico; § Leibniz Institute for Vegetable and Ornamental Crops (IGZ) e.V., Theodor-Echtermeyer-Weg 1, 14979, Großbeeren, Germany; ∥ Institute of Biodiversity, Friedrich Schiller University Jena, Dornburger-Str. 159, 07743, Jena, Germany

## Abstract

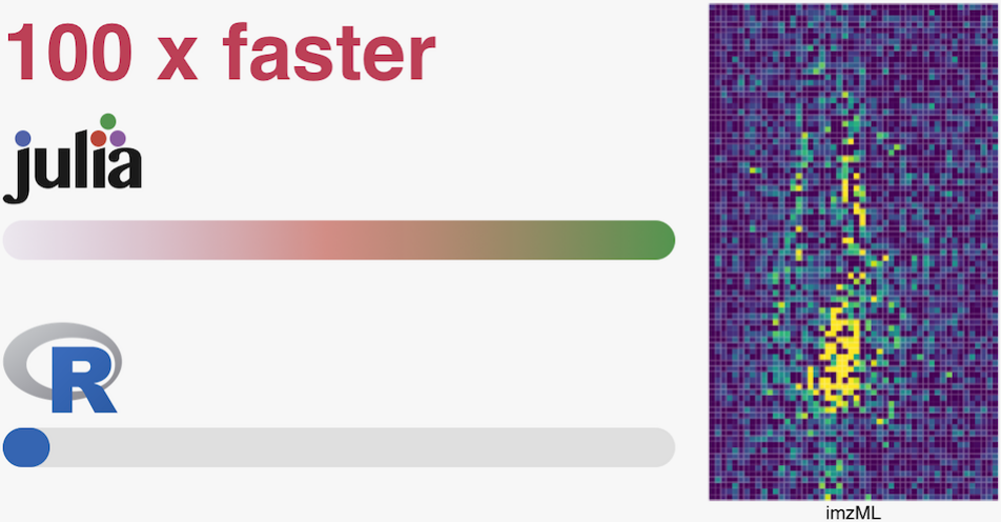

Julia combines the virtues of high-level and low-level programming languages: The code is human-readable, and the performance of the created binaries competes with machine-orientated compilers. Thus, Julia is popular in “Big Data” sciences. Reading mass spectrometry (MS) data with Julia was impossible until now due to missing libraries. Here, we present a Julia library for importing mass spectrometry (MS) data in HUPO standard mzML and imzML formats and demonstrate its function with direct and ambient ionization MS, liquid chromatography-MS, and MS imaging data on standard platforms (Windows, Linux, and Mac OS). The processing speed of Julia for reading imzML MS imaging files was up to 214 times faster than the comparable code in R. Julia can remove bottlenecks for computationally demanding tasks in large-scale MS-Omics and MS imaging data processing workflows and supports their agile development. In addition, time-critical and complex data evaluation tasks become possible, such as following the real-time monitoring of biological processes and pattern recognition in large MS imaging projects. Our mzML/imzML libraries and code examples are available under the terms of the MIT license from https://github.com/CINVESTAV-LABI/julia_mzML_imzML.

## Introduction

Mass spectrometry (MS) can analyze complex mixtures of chemical compounds with high sensibility and selectivity; thus, MS is a standard method in science and industries. Nonetheless, high resolution and fast scanning speeds lead to large MS data files that require adequate processing and interpretation.

For standard tasks, such as the statistical evaluation of features and the identification of molecules or proteomic workflows, the providers of MS equipment offer computationally efficient and easy-to-use software.

Yet, particular research questions and novel MS methods, such as ambient sources with unusual ionization mechanisms, require manual data processing and the coding of new software. As a result, several programming languages have been adopted for MS data analysis:^1^ so-called “interpreters” that process the code line-wise on the host computer, and “compilers” that translate the complete program code to platform-dependent binaries before execution. Generally, interpreting languages are high-level, relatively easy to code and debug, and portable but slow. Conversely, compilers produce platform-optimized and efficient programs, but the source code is often complex to read and modify.

Currently, the interpreter R (https://R-project.org)^2^ is very popular in the MS community: several packages and scripts facilitate the MS data processing,^3,4^ statistical analyses^5−8^ and visualization.^9^ R is relatively easy to program and well-documented. Besides, a huge community provides help and support. On the downside, R is slow and consumes many computational resources. Using multiple CPUs requires additional code and memory,^10^ making the use of R less attractive for large data sets.

Python (https://python.org) is computationally more efficient than R, user-friendly, and can be used, for example, to create raw data processing and proteomic workflows.^11^ In addition, the Python toolkits such as tk facilitate the fast development of programs with a graphical user interface.^12,13^ Nevertheless, Python is less used in the MS community and as an interpreter language, i.e., relatively slow.

Programs written in C (https://www.iso.org/standard/74528.html) and C++ (https://cplusplus.com/) are fast and memory-efficient and thus ideal for processing large data sets. Projects such as ProteoWizard and OpenMS also provide code examples and open libraries for developing new MS programs with C++. Some programs use C/C++ code for time-critical calculations.^14,15^ Besides the more complex syntax, compiling and linking the executable binary may be challenging, which is why C/C++ programs are primarily used by experts.

Java (https://www.java.com) is a compiling language for creating platform-independent programs and is used, for example, in mzMine.^16−19^ Java code is “byte compiled” and requires a Java Virtual Maschine (JVM), which is part of the Java Runtime Environment (JRE) for running Java programs on the host computer.

In 2023, the “Rusteomics” project (https://github.com/rusteomics) was started by the European Bioinformatics Community for Mass Spectrometry (EuBIC-MS) and works on the development of a toolbox for proteomics and mass spectrometry using the Rust language. The programming language Julia (https://julialang.org) was first presented on Valentine’s Day 2012. It was designed from scratch as a “Goldilocks” language that combines the advantages of high-level languages such as R and low-level languages such as C.^20^ The syntax is similar to interpreting languages such as R and Python, but the code is compiled in the first run. This concept is called “ahead-of-time” (AOT) or “just-in-time” (JIT) compilation.^21^ Early adopters from R experienced a drastically faster execution of their programs, resulting in a fast-growing community.^20^

A Case Study webpage (https://juliahub.com/case-studies) demonstrates the use of Julia in numerically demanding areas such as astronomy and basic physics, drug development, and energy network simulations. For example, Nobel Laureate Thomas J. Sargent uses Julia for macroeconomic modeling.

Up until now, libraries for importing and processing community-format mzML and imzML files MS data, have been missing in Julia. Here, we report a Julia library for reading mzML and imzML data and test the processing of diverse MS and MS imaging data sets.

## Experimental Section

### HUPO Mass Spectrometry Standards

The *Proteomics Standards Initiative* (PSI) working group of the Human Proteome Organization (HUPO) develops data formats to facilitate data comparison, verification, and exchange. The mzML standard defines an open XML-based format for mass spectrometry data.^22^

XML is an acronym for *Extensible Markup Language*, a text-based format for encoding hierarchically structured information. The XML format offers human readability. XML also supports Unicode, which allows the storage of data in any human language; moreover, a text-based format is both platformand programming-language-independent, providing long-time compatibility.

The human-readable information stored inside an XML file needs a hierarchical binary data structure transformation suitable for the analysis software functions; this process is termed *parsing*. Some popular XML parser libraries are Microsoft Core XML Services (MSXML), Saxon, System.Xml.XmlDocument, and Xerces.

In this work, we implemented a dedicated lightweight parsing software. The parser finds predefined XML tags that define the scope of an XML element. Once the parser matches a target tag, its numerical *value* is extracted. A widespread method for programming complex string searches is the Regular Expression (Regex) engine. Many modern programming languages, including Julia, support Regex operations natively.

### Library Development and Functions

#### mzML Format and Loading

The mzML format has an optional feature for allowing random spectra access. The presence of <indexedmzML> as the top element identifies an indexed mzML document; the mzML information itself is followed by a tag named <indexList> where byte-based offsets for random spectra data access are stored. A file pointer offset element called <indexListOffset> is located near the end of the file and contains the file offset of the <indexList> element. Although the indexed information is not mandatory, it seems to have been adopted by most mzML writing applications; therefore, this work assumes file index availability to prevent unnecessary string searches.

Loading an mzML file requires a call to the library function LoadMzml, passing the full file name as a parameter. This function opens the file with read-only attributes, looking for the indexListOffset element in the final portion of the file to gain access to the spectrum offset list, whose content is loaded by a helper function termed ParseOffsetList.

Each offset in the list points to an <spectrum> element, with several child elements describing the axes’ labels, their units, and the instrument configuration. This additional information is mandatory for signal processing algorithms and can be safely omitted. For MS data processing, the <binaryDataArrayList> subelement defines the data type and its compression schema. The attribute value MS:1000514 of the <cvParam> child element identifies the *m*/*z* axis, and the string MS:1000515 the intensity values. The spectral information loading happens inside the function <LoadSpectra>.

As spectral data is predominantly binary, the standardization group decided to encode binary data as ASCII strings. MIME is the short-term for Multipurpose Internet Mail Extensions, an Internet standard for supporting multimedia inside ASCII-based email files; in particular, the base64 encoding scheme was adopted for mzML binary data storage. Binary packages could be compressed with the Zlib compression algorithm. Deflating compressed data packages is made through Libz Julia’s library; this is the only external dependence of our mzML/imzML library. The ReadVector function handles the decoding and extracting *m*/*z* and intensity vectors stored inside the <binary> subelements.

The spectral axis storage does not follow a fixed order: both intensity and *m*/*z* can appear as the content of the first <binaryDataArray> element. However, during our test, mzML files always kept the same axis order within the entire file. Thus, the LoadMzml function only determines the axis order in the first spectrum and applies the same reading sequence in every file spectrum.

The library defines a data structure SpecDim. Its principal purpose is storing the axis order sequence, axis data type, and packing schema. The helper function ConfigSpecDim decodes and loads the information from the first spectrum in the file. To reduce unnecessary text parsing, the “SpecDim” function also defines the field Skip, which contains a count of the bytes that can be ignored. The count starts in the <spectrum> tag up to its corresponding <binaryDataArrayList> subelement.

Finally, the spectral information is stored as a numeric array, where each cell array contains a two-row column matrix with the *m*/*z* values stored in the first column and the intensity scans stored in the second column.

#### imzML Format and Loading

The imzML format for mass spectrometry imaging (MSI) data was presented in 2011.^23^ One of the main reasons for not using the mzML format for the imzML image storage is to improve the reading performance. Spectral data is stored in an external binary file to handle large data sets efficiently. Thus, two files are necessary for storing mass spectrometry imaging (MSI) data: The first has the imzML extension and contains the properties of each stored spectrum in XML format; the ibd file holds the spectral information on each pixel stored as a binary stream.

Despite this limitation, the imzML format is fully compatible with version 1.1 of the mzML standard. The <binary> subelements are now empty, but new <vcParam> mapping rules were introduced for locating the offsets of each spectrum comprising the image. Given the compatibility between mzML and imzML formats, some functions of the mzML parsing code are reused when parsing both formats. The shared functions are part of the Common.jl library file.

Our imzML library assumes the so-called *processed format* where each spectrum can have a distinct data length and axis values.

The adopted strategy for loading *m*/*z* image information starts with the <referenceableParamGroup> subelement decoding, where the axis data type description is stored. The decoding action happens inside the AxesConfigImg function, which returns the axis configuration as the SpecDim structures previously described.

Image dimensions are parsed next, employing the GetImgDimensions function. The accession property of the subelement <cvParam> contains the maximal pixel count of the *x* and *y* axis. Sometimes, the number of pixels stored in the file does not correspond with the product of the image dimensions. The count property of the <spectrumList> subelement holds the correct number of spectral pixels in the file. This variable is fundamental for memory allocation and spectral load loop control. Each <spectrum> element in the imzML format contains information on the pixel coordinates. The GetSpectrumAttributes function decodes the tag <scan>, looking for the accession values of the <cvParam> subelement, namely IMS:1000051 and IMS:1000051, which stores the *x* and *y* pixel position. In addition, the <cvParam> accessions IMS:1000102 and IMS:1000103 of the <referenceableParamGroupRef> tag have to be decoded since they contain the file offset where the axis data values are stored, and the axis element count.

The approximate skip-byte counts are computed for each of the aforementioned accession properties. Inside the spectrum read loop, which occurs inside the LoadImgData function, the byte count is added to the current access file pointer; therefore, the next load text operation is very close to the character sequence that defines the pixel coordinates. After decoding the accession of interest, the file pointer is updated with the next skip-byte count, omitting many irrelevant characters and improving the reading performance.

The output data structure resulting from the call to the LoadImzml function is a vector, where each element is another vector list with the *x* image coordinate stored in its first element, the *y* image coordinate in the second, a vector with the *m*/*z* axis values in the third element, and its corresponding intensity stored in the fourth element.

### Data Sets

For testing the Julia library, we used three mzML and four imzML mass spectrometry data sets:

- **Col_1.mzML** is a liquid chromatography (LC) ESI MS data set from an *Arabidopsis* extraction.^24^
- **Cytochrome_C.mzML** is an electrospray mass spectrometry (ESI MS) data set of Cytochrome C.^12^
- **T9_A1.mzML** is a low-temperature plasma (LTP) MS data set of the interaction between *Arabidopsis* and Trichoderma.^25^
- **imzML_AP_SMALDI.zip** contains an AP-SMALDI mass spectrometry imaging data set of mouse urinary bladder slides.^26,27^
- **imzML_DESI.zip** is a DESI mass spectrometry imaging data set of human colorectal cancer tissue.^28^
- **imzML_LA-ESI.zip** is an LA-ESI mass spectrometry imaging data set of an *Arabidopsis thaliana* leaf.^29^
- **imzML_LTP.zip** was generated by low-temperature plasma ionization ambient mass spectrometry imaging of a chili fruit.^30,31^

The test data are available from Zenodo: 10.5281/zenodo.10084132.

Table 1 lists the data sets used for testing our Julia library’s compatibility and computational performance with mass spectrometry imaging (MSI) files.

**Table 1:** Mass spectrometry imaging data sets. AP-MALDI Atmospheric Pressure Matrix Assisted Laser Desorption/Ionization, DESI Desorption Electrospray Ionization, LAESI Laser Ablation Electrospray Ionization, LTP Low-temperature Plasma

set	organism, tissue	technique	res. [μm]	pixels	spectra	size [MB]
1	Mouse, urinary bladder	AP-MALDI (+)	10	260 × 134	34,860	833
2	Human, colorectal cancer	DESI (−)	100	67 × 64	4,288	610
3	Chili, fruit	LTP (+)	1000	85 × 50	4,250	552
4	*A. thaliana*, leaf	LAESI (−)	200	46 × 26	1,196	365

### Computers and Software

The example programs were tested on Windows, MacOS, and Linux operating systems, using consumer-grade computers with 4 to 16 CPU cores and 16 or 32 GiB RAM.

We used VSCode version 1.84, R version 4.3, and Julia version 1.9 to edit and run the code.

### Code Availability and License

The Julia library and example programs are available from https://github.com/CINVESTAV-LABI/julia_mzML_imzML under the terms of the MIT license.

## Results

To test the Julia library, we reanalyzed published data sets listed in the Experimental Section.

### Analysis of MS and LC-MS Data Sets

**Figure 1:**
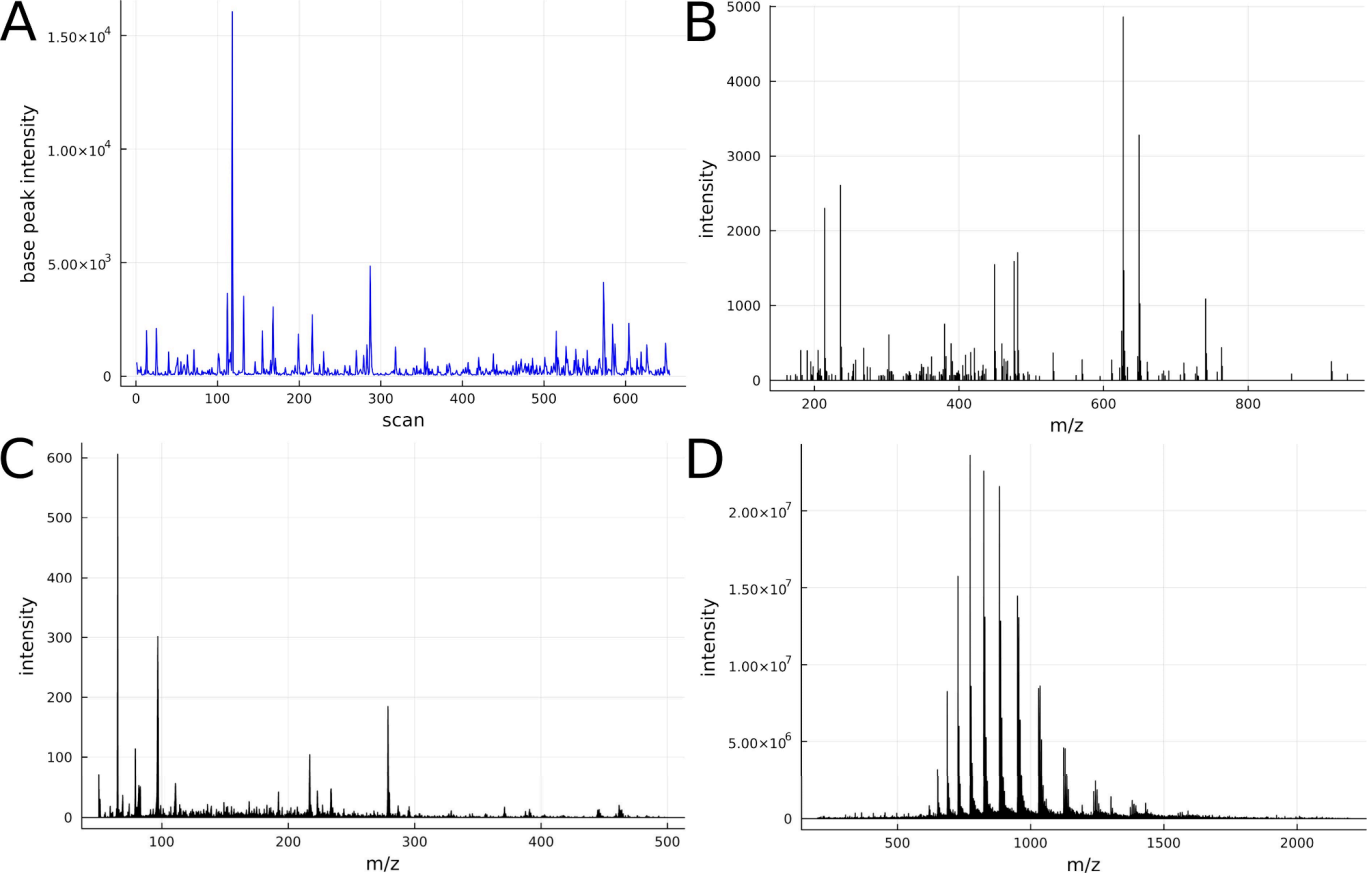
A) Base-Peak Chromatogram (BPC) of LC-MS data from plant metabolomics (*Arabidopsis thaliana* extract); B) MS scan from LC-MS run shown in A; C) Spectrum from ambient ionization mass spectrometry (plant-fungal interaction between *Arabidopsis thaliana* and Trichoderma atroviride, monitored by low-temperature plasma MS); D) ESIMS spectrum of cytochrome C.

Figure 1A displays a plant metabolomics study’s Base-Peak Chromatogram (BPC). The extracts of *Arabidopsis thaliana* were analyzed by liquid-chromatography, coupled to a highresolution qToF mass analyzer.^24^ The LC-MS data were denoised and centroided using msconvert from the ProteoWizard project.^32^ The BPC and a random single scan, shown in Figure 1B, are similar to the results obtained by data processing with R.^33^

The processing of ambient ionization mass spectrometry (AIMS) data often requires the development of custom workflows and software. Figure 1C shows a scan from the *in vivo* monitoring of a plant-fungal interaction with low-temperature plasma ionization MS. Using Julia, we performed time-series analyses (autocorrelations and Poincaré plots), demonstrating the role of 6-pentyl-α-pyrone (6-PP) in the homeostasis between *Trichoderma atroviride* and *Arabidopsis thaliana*.^25^

Figure 1D derives from the electrospray ionization of cytochrome C and presents multicharged protein ions. The data were generated on a low-resolution ion trap and published in 2010.^12^

Following the Unix principle of a minimal and modular software design, we did not include further spectrum processing or feature detection functions in our Julia library. However, the necessary unit operations can be programmed with few lines. For example, loading the LC-MS data set and generating and plotting the BPC with axes labels into a PDF file only needs five lines of code:

# load mzML spectra
spectra = LoadMzml(“Col_1.mzML”)

# create Base Peak Chromatogram Plot (BPC) in blue color
mz = plot(maximum(spectra[2,:]), lc=:blue, legend=false)
xlabel!(“scan”)
ylabel!(“base peak intensity”)
savefig(mz, “Col_1_BPC.pdf”)

The resulting plot is displayed in Figure 1A.

### Analysis of Mass Spectrometry Imaging Data

All four imzML mass spectrometry imaging (MSI) listed in Table 1 were successfully loaded into Julia using our library.

These data sets were generated with different methods, mass analyzers, and software from both commercial and development platforms, thus demonstrating the robustness and broad compatibility of our Julia library for reading imzML files.

The human perception of colors needs to be respected for visualizing MSI signal intensities. For example, rainbow color maps that are still frequently used lead to a wrong impression of the abundance of a signal or compound.^34^ Therefore, we use the “viridis” color map to represent signal intensities correctly.^35^

The visualization of MSI data is often hampered by noise signals that obscure local differences. Therefore, we developed the “*Threshold Intensity Quantization*” (TrIQ) algorithm that reduces noise, optimizes the contrast, and maintains the true signal intensities.^36^ We implemented the TrIQ data set into the Julia library, and bitmap figures of *m*/*z* intensity distributions can be created with two code lines:

# Extract image slice data
slice = GetSlice(spectra, 885.55, 0.005)

# Save image, using the TrIQ algorithm and the Viridis color map
SaveBitmap(“TrIQ.bmp”, TrIQ(slice, 256, 0.95), ViridisPalette)

**Figure 2:**
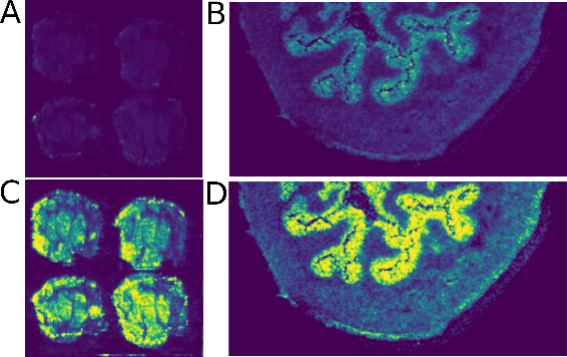
Visualization of mass spectrometry imaging data with Julia Subfigures A) and B) representing the raw ion intensities with a Viridis color map. Subfigures C) and D) used the TrIQ algorithm for contrast optimization. A) and C): MSI data set 2; B) and D): MSI data set 1, see Table 1.

Figure 2 demonstrates the effect of the TrIQ algorithm on the mouse urinary bladder and human colorectal cancer MSI data sets.

### Computational Performance

We evaluated the computational performance of Julia in comparison to R by calculating the average time for loading the mass spectrometry imaging (MSI) data sets of Table 1 ten times. The R and Julia test scripts are included in the code repository. We tested macOS, Ubuntu Linux, and two Microsoft Windows operating systems on consumer-grade laptops.

**Figure 3:**
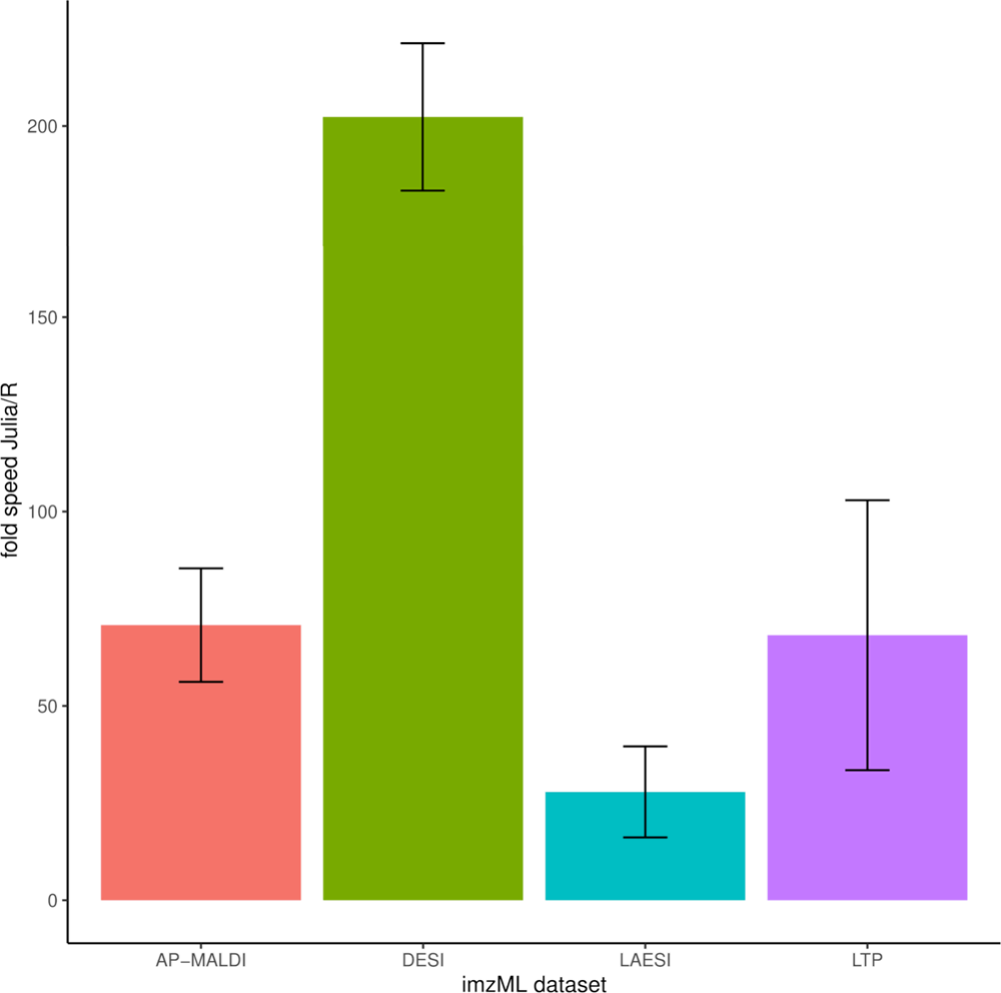
Loading times for imzML data sets on different operating systems. The data sets are listed in Table 1.

As shown in Figure 3, loading MSI data was always faster with Julia, compared to R, independently of the used hardware and operating system. On average, the Julia code was 92 times faster than the comparable R code. The least speed increase was 17-fold, and the highest was 214 fold.

The absolute loading times (average of ten repetitions) were between 0.1 and 2.3 s. Twelve combinations were tested; in only three cases, the loading of an MSI data set took more than a second. No computer system was consistently slower or quicker than the others.

## Discussion

A typical workflow for mass spectrometry data analysis consists of five steps:^1,37^ 1) Raw data import, 2) spectra processing, 3) feature analysis, 4) statistics and data mining, and 5) model building and interpretation.

There are numerous excellent tools for the statistical evaluation of MS features, metabolic network reconstruction, etc. (steps 4–5). For example, the MetaboAnalyst Web server https://www.metaboanalyst.ca/ provides a complete platform for metabolomics-based systems biology, including machine learning, biomarker search, etc.^5,8^ The free statistical computing and visualization language R provides many data analysis and mining packages.^2,38^ Therefore, R was also adopted for processing mass spectrometry data, and several libraries allow the direct import of raw files.^3,4,7,9,39^

Regardless, the first steps (1–3) of MS data processing are computationally demanding. A first optimization of workflows is possible by adjusting the conversion of raw binary instrument data to mzML. The ProteoWizard tools^32^ can be used for efficient noise reduction, centroiding, etc., and trim the file sizes without losing spectral information relevant to the analytical question.^24^

Loading the MS data into an object, such as an array, is the bottleneck for most custom workflows and can take minutes for large data sets, even on state-of-the-art computers. In addition, the further handling of large objects might be limited in interpreting languages such as R because they are not optimized for computational performance. For example, R scripts for processing mass spectrometry imaging (MSI) data use only one CPU by default.^10^ With Julia, the loading of MSI data was 1 or 2 orders of magnitude faster than R (Figure 3). Even with consumer-grade computers, imzML data sets were loaded in fractions of seconds. We also demonstrated the compatibility of our library with typical MS data sets, e.g., from ambient ionization MS and liquid-chromatography coupled to MS (see section Analysis of MS and LC-MS Data Sets).

In addition, using the library and implementing advanced algorithms, such as the Thresh-old Intensity Quantitation algorithm *TrIQ* (see section Analysis of Mass Spectrometry Imaging Data) for the contrast optimization of MSI graphics, is facile.

Consequently, Julia functions can optimize existing workflows by replacing slow unit operations such as data loading and preprocessing. Further, Julia can speed up the programming testing cycles in workflow development or for designing complete Julia data processing pipelines for mass spectrometry data. Thus, the Julia libraries contribute to the existing ecosystem of open mass spectrometry software.

## Conclusions

We provide the library for reading HUPO mzML and imzML mass spectrometry data with Julia. Julia’s high computational performance, cross-platform compatibility, and intuitive programming pave the road for analyzing massive mass spectrometry (MS) data, e.g., from MS Omics and mass spectrometry imaging (MSI).

Our tests demonstrated compatibility with different types of MS and MSI data; loading MSI data was up to 2 orders of magnitude faster than a similar script in R. Thus, Julia can support the agile development of workflows and replace code currently slowing down MS data processing pipelines. Julia is especially suitable for time-critical applications like real-time volatilomics and “Big Data” mining, such as pattern recognition in MSI projects.

Our library only contains basic functions for reading mzML and imzML files with Julia. Following the Clean Code philosophy, we will focus further development on stability, robustness, and efficiency rather than quickly implementing new features. Yet, depending on the feedback from the community, we will consider implementing basic unit operations, such as peak picking and feature alignment, in the future.
